# Successful complete closure of anastomotic leak in ileal pouch anal anastomosis with endoscopic helix tacking device

**DOI:** 10.1055/a-2795-8023

**Published:** 2026-02-24

**Authors:** Eleni Nakou, Panagiotis Dikeakos, Marianna Spinou, Dimitrios Dimitriadis, Petros Zormpas, Margarita-Eleni Manola, George Tribonias

**Affiliations:** 1 168201 Gastroenterology Department, General Hospital Korgialenio-Benakio Red Cross, Athens, Greece; 2 69185 First Surgical Department, Geniko Nosokomeio Peiraia Tzaneio, Pireas, Greece

Anastomotic leaks remain a serious complication after ileal pouch–anal anastomosis (IPAA), ranging from acute events to chronic leaks leading to pouch dysfunction or failure 1 . Although traditionally managed surgically, minimally invasive endoscopic approaches are increasingly used, with variable success rates 1 2 .

We report the first successful endoscopic closure of an anastomotic leak using a suturing device after ileoanal pouch construction.

A 53-year-old man with ulcerative colitis and prior laparoscopic subtotal colectomy was admitted for ileoanal pouch construction. The procedure included end ileostomy takedown, extensive adhesiolysis, transabdominal and transanal rectal stump excision, construction of a 16-cm ileoanal pouch and creation of a protective loop ileostomy.

On the 5th postoperative day, the patient developed fever and purulent output from the surgical drain. Computed tomography demonstrated an anastomotic leak and exploratory laparotomy was performed identifying the site of rupture in the IPAA. Thorough lavage of the peritoneal cavity was performed and surgical drains were placed in all abdominal compartments.

**Fig. 1:**
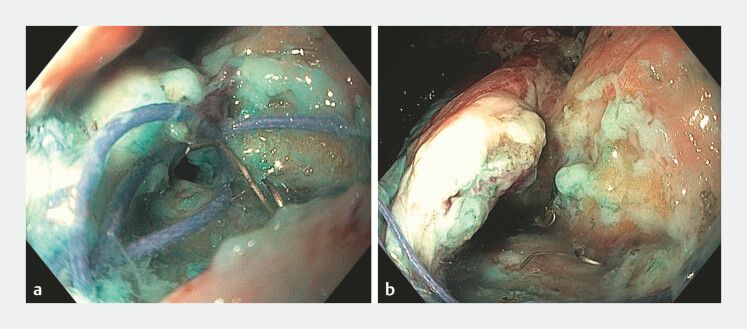
**a** A close-up endoscopic view of a 20-mm anastomotic leak at the pouch–anal anastomosis. **b** A wide-field endoscopic view of a 20-mm anastomotic leak at the pouch–anal anastomosis.

The patient demonstrated no signs of peritonitis and conservative management was chosen. On the 12th postoperative day, the patient was referred to the gastroenterology department for the endoscopic management of the leak. During endoscopy, a leak of approximately 20 mm in diameter was identified near the dentate line at the anal–pouch anastomosis ( Fig. 1 **a, b** ). Endoscopic complete closure with a helix tacking device (X-Tack, Boston Scientific) was performed successfully ( Video 1 ).

Endoscopic management of an IPAA anastomotic leak. The video demonstrates the identification of the defect and complete closure using the helix tacking device (X-Tack), followed by surveillance endoscopy at 4 and 12 months. IPAA, ileal pouch–anal anastomosis.Video 1

**Fig. 2:**
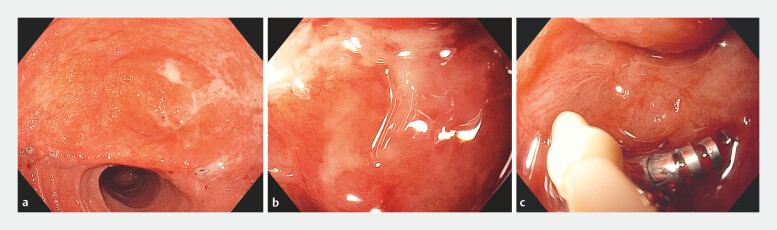
**a** A wide-field endoscopic view demonstrating complete mucosal healing at an 18-month follow-up. **b** A close-up endoscopic view of the healed mucosa at an 18-month follow-up. **c** An X-Tack device in situ at an 18-month follow-up.

The drain output decreased and the patient was discharged in good condition. Follow-up endoscopy at 4, 12 and 18 months demonstrated complete mucosal healing ( Fig. 2 **a, b** ) with the X-Tack device in situ ( Fig. 2 **c** ). The patient remained asymptomatic through the 18-month follow-up and it was decided to proceed with takedown of the loop ileostomy.

Endoscopic suturing devices offer a promising minimally invasive option for managing postoperative complications after IPAA, potentially reducing morbidity and reoperation challenges.

Endoscopy_UCTN_Code_CPL_1AJ_2AJ
